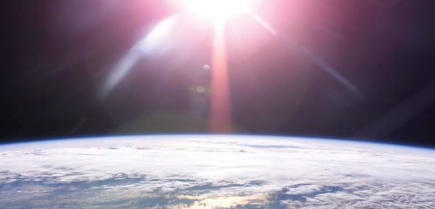# Benefits of Sunlight: A Bright Spot for Human Health

**DOI:** 10.1289/ehp.116-a160

**Published:** 2008-04

**Authors:** M. Nathaniel Mead

Each day, Apollo’s fiery chariot makes its way across the sky, bringing life-giving light to the planet. For the ancient Greeks and Romans, Apollo was the god of medicine and healing as well as of sun and light—but Apollo could bring sickness as well as cure. Today’s scientists have come to a similarly dichotomous recognition that exposure to the ultraviolet radiation (UVR) in sunlight has both beneficial and deleterious effects on human health.

Most public health messages of the past century have focused on the hazards of too much sun exposure. UVA radiation (95–97% of the UVR that reaches Earth’s surface) penetrates deeply into the skin, where it can contribute to skin cancer indirectly via generation of DNA-damaging molecules such as hydroxyl and oxygen radicals. Sunburn is caused by too much UVB radiation; this form also leads to direct DNA damage and promotes various skin cancers. Both forms can damage collagen fibers, destroy vitamin A in skin, accelerate aging of the skin, and increase the risk of skin cancers. Excessive sun exposure can also cause cataracts and diseases aggravated by UVR-induced immunosuppression such as reactivation of some latent viruses.

However, excessive UVR exposure accounts for only 0.1% of the total global burden of disease in disability-adjusted life years (DALYs), according to the 2006 World Health Organization (WHO) report *The Global Burden of Disease Due to Ultraviolet Radiation*. DALYs measure how much a person’s expectancy of healthy life is reduced by premature death or disability caused by disease. Coauthor Robyn Lucas, an epidemiologist at the National Centre for Epidemiology and Population Health in Canberra, Australia, explains that many diseases linked to excessive UVR exposure tend to be relatively benign—apart from malignant melanoma—and occur in older age groups, due mainly to the long lag between exposure and manifestation, the requirement of cumulative exposures, or both. Therefore, when measuring by DALYs, these diseases incur a relatively low disease burden despite their high prevalence.

In contrast, the same WHO report noted that a markedly larger annual disease burden of 3.3 billion DALYs worldwide might result from very low levels of UVR exposure. This burden subsumes major disorders of the musculoskeletal system and possibly an increased risk of various autoimmune diseases and life-threatening cancers.

The best-known benefit of sunlight is its ability to boost the body’s vitamin D supply; most cases of vitamin D deficiency are due to lack of outdoor sun exposure. At least 1,000 different genes governing virtually every tissue in the body are now thought to be regulated by 1,25-dihydroxyvitamin D_3_ (1,25[OH]D), the active form of the vitamin, including several involved in calcium metabolism and neuromuscular and immune system functioning.

Although most of the health-promoting benefits of sun exposure are thought to occur through vitamin D photosynthesis, there may be other health benefits that have gone largely overlooked in the debate over how much sun is needed for good health [see “Other Sun-Dependent Pathways,” p. A165]. As for what constitutes “excessive” UVR exposure, there is no one-size-fits-all answer, says Lucas: “‘Excessive’ really means inappropriately high for your skin type under a particular level of ambient UVR.”

## Vitamin D Production

Unlike other essential vitamins, which must be obtained from food, vitamin D can be synthesized in the skin through a photosynthetic reaction triggered by exposure to UVB radiation. The efficiency of production depends on the number of UVB photons that penetrate the skin, a process that can be curtailed by clothing, excess body fat, sunscreen, and the skin pigment melanin. For most white people, a half-hour in the summer sun in a bathing suit can initiate the release of 50,000 IU (1.25 mg) vitamin D into the circulation within 24 hours of exposure; this same amount of exposure yields 20,000–30,000 IU in tanned individuals and 8,000–10,000 IU in dark-skinned people.

The initial photosynthesis produces vitamin D_3_, most of which undergoes additional transformations, starting with the production of 25-hydroxyvitamin D (25[OH]D), the major form of vitamin D circulating in the bloodstream and the form that is routinely measured to determine a person’s vitamin D status. Although various cell types within the skin can carry out this transformation locally, the conversion takes place primarily in the liver. Another set of transformations occurs in the kidney and other tissues, forming 1,25(OH)D. This form of the vitamin is actually a hormone, chemically akin to the steroid hormones.

1,25(OH)D accumulates in cell nuclei of the intestine, where it enhances calcium and phosphorus absorption, controlling the flow of calcium into and out of bones to regulate bone-calcium metabolism. Michael Holick, a medical professor and director of the Bone Health Care Clinic at Boston University Medical Center, says, “The primary physiologic function of vitamin D is to maintain serum calcium and phosphorous levels within the normal physiologic range to support most metabolic functions, neuromuscular transmission, and bone mineralization.”

Without sufficient vitamin D, bones will not form properly. In children, this causes rickets, a disease characterized by growth retardation and various skeletal deformities, including the hallmark bowed legs. More recently, there has been a growing appreciation for vitamin D’s impact on bone health in adults. In August 2007, the Agency for Health Care Policy and Research published *Effectiveness and Safety of Vitamin D in Relation to Bone Health*, a systematic review of 167 studies that found “fair evidence” of an association between circulating 25(OH)D concentrations and either increased bone-mineral density or reduced falls in older people (a result of strengthened muscles as well as strengthened bones). “Low vitamin D levels will precipitate and exacerbate osteoporosis in both men and women and cause the painful bone disease osteomalacia,” says Holick.

## Evolution of the Great Solar Debate

In the 2002 book *Bone Loss and Osteoporosis in Past Populations: An Anthropological Perspective*, Reinhold Vieth, a nutrition professor at the University of Toronto, writes that early primates probably acquired their relatively high vitamin D requirements from frequent grooming and ingestion of oils rich in vitamin D precursors that were secreted by their skin onto their fur. The first humans evolved in equatorial Africa, where the direct angle of sunlight delivers very strong UVR most of the year. The gradual loss of protective fur may have created evolutionary pressure to develop deeply pigmented skin to avoid photodegradation of micronutrients and protect sweat glands from UVR-induced injury.

In the July 2000 issue of the *Journal of Human Evolution*, California Academy of Sciences anthropologists Nina Jablonski and George Chaplin wrote that because dark skin requires about five to six times more solar exposure than pale skin for equivalent vitamin D photosynthesis, and because the intensity of UVB radiation declines with increasing latitude, one could surmise that skin lightening was an evolutionary adaptation that allowed for optimal survival in low-UVR climes, assuming a traditional diet and outdoor lifestyle. Cooler temperatures in these higher latitudes resulted in the need for more clothing and shelter, further reducing UVR exposure. With shorter winter days and insufficient solar radiation in the UVB wavelengths needed to stimulate vitamin D synthesis, dietary sources such as fatty fish became increasingly important.

Over time, clothing became the norm in higher latitudes and then eventually a social attribute in many societies. By the 1600s, peoples in these regions covered their whole body, even in summertime. Many children who lived in the crowded and polluted industrialized cities of northern Europe developed rickets. By the late 1800s, approximately 90% of all children living in industrialized Europe and North America had some manifestations of the disease, according to estimates based on autopsy studies of the day cited by Holick in the August 2006 *Journal of Clinical Investigation* and the October 2007 *American Journal of Public Health*.

Doctors throughout Europe and North America began promoting whole-body sun-bathing to help prevent rickets. It was also recognized that wintertime sunlight in the temperate zone was too feeble to prevent rickets. For this reason, many children were exposed to UVR from a mercury or carbon arc lamp for one hour three times a week, which proved to be an effective preventive measure and treatment.

Around the time the solar solution to rickets gained widespread traction in medical circles, another historic scourge, tuberculosis (TB), was also found to respond to solar intervention. TB patients of all ages were sent to rest in sunny locales and generally returned in good health. Dermatology professor Barbara A. Gilchrest of Boston University School of Medicine says that, whereas sun exposure was shown to improve cutaneous TB, sanatorium patients with pulmonary TB likely responded as much or more to rest and good nutrition than to UVR. Nevertheless, a meta-analysis published in the February 2008 *International Journal of Epidemiology* found that high vitamin D levels reduce the risk of active TB (i.e., TB showing clinical symptoms) by 32%.

Almost overnight, as awareness of the sun’s power against rickets and TB spread, attitudes toward sun exposure underwent a radical shift. The suntan became valued in the Western world as a new status symbol that signified both health and wealth, as only the affluent could afford to vacation by the sea and play outdoor sports. Phototherapy quickly emerged as a popular medical treatment not only for TB, but also for rheumatic disorders, diabetes, gout, chronic ulcers, and wounds. The “healthy tan” was in, and “sickly-looking” pale skin was out.

## Cancer: Cause, Protection, or Both?

The first reports of an association between sun exposure and skin cancer began to surface in dermatology publications in the late nineteenth century. Nevertheless, it was not until the 1930s that the U.S. Public Health Service began issuing warnings about sun-related health risks. People were cautioned to avoid the midday summer sun, cover their heads in direct sunlight, and gradually increase the time of sun exposure from an initial 5–10 minutes per day to minimize the risk of sunburn.

In the decades that followed, the skin cancer hazards of excessive sun exposure would be extensively studied and mapped. Today, the three main forms of skin cancer—melanoma, basal cell carcinoma, and squamous cell carcinoma—are largely attributed to excessive UVR exposure. Skin cancers became the most common form of cancer worldwide, especially among groups such as white residents of Australia and New Zealand.

When atmospheric scientists first called attention to possible chemical destruction of the stratospheric ozone layer in the early 1970s, one predicted consequence of the increased UVB radiation was a rise in skin cancer rates, especially in Australia, New Zealand, South Africa, and Latin America. To counter this threat, the WHO, the United Nations Environment Programme, the World Meteorological Organization, the International Agency for Research on Cancer, and the International Commission on Non-Ionizing Radiation Protection established INTERSUN, the Global UV Project, with the express goal of reducing the burden of UVR-related disease. INTERSUN activities have included the development of an internationally recognized UV Index to help frame sun protection messages related to the daily intensity of UVR. [For more information on these activities, see “WHO Ultraviolet Radiation Website,” p. A157 this issue.]

Australia was among the first countries to spearhead large-scale sun protection programs, with the Slip-Slop-Slap initiative (short for “slip on a shirt, slop on some sun-screen, and slap on a hat”) introduced in the early 1980s. “This program and the subsequent SunSmart campaign have been highly effective in informing Australians of the risks and providing clear, practical instructions as to how to avoid excessive UVR exposure,” says Lucas. As a result of increased use of hats, sunscreen, and shade, the incidence of malignant melanoma has begun to plateau in Australia, New Zealand, Canada, and Northern Europe among some age groups. However, because other UVR-induced skin cancers typically take longer than melanoma to develop, their incidence rates continue to rise in most developed countries. Lucas says a gradual improvement in these rates is to be expected as well.

Whereas skin cancer is associated with too much UVR exposure, other cancers could result from too little. Living at higher latitudes increases the risk of dying from Hodgkin lymphoma, as well as breast, ovarian, colon, pancreatic, prostate, and other cancers, as compared with living at lower latitudes. A randomized clinical trial by Joan Lappe, a medical professor at Creighton University, and colleagues, published in the June 2007 issue of the *American Journal of Clinical Nutrition*, confirmed that taking 2–4 times the daily dietary reference intake of 200–600 IU vitamin D_3_ and calcium resulted in a 50–77% reduction in expected incidence rates of all cancers combined over a four-year period in post-menopausal women living in Nebraska.

Moreover, although excessive sun exposure is an established risk factor for cutaneous malignant melanoma, continued high sun exposure was linked with increased survival rates in patients with early-stage melanoma in a study reported by Marianne Berwick, an epidemiology professor at the University of New Mexico, in the February 2005 *Journal of the National Cancer Institute*. Holick also points out that most melanomas occur on the least sun-exposed areas of the body, and occupational exposure to sunlight actually reduced melanoma risk in a study reported in the June 2003 *Journal of Investigative Dermatology*.

## Other Health Links

Various studies have linked low 25(OH)D levels to diseases other than cancer, raising the possibility that vitamin D insufficiency is contributing to many major illnesses. For example, there is substantial though not definitive evidence that high levels of vitamin D either from diet or from UVR exposure may decrease the risk of developing multiple sclerosis (MS). Populations at higher latitudes have a higher incidence and prevalence of MS; a review in the December 2002 issue of *Toxicology* by epidemiology professor Anne-Louise Ponsonby and colleagues from The Australian National University revealed that living at a latitude above 37° increased the risk of developing MS throughout life by greater than 100%.

Still to be resolved, however, is the question of what levels of vitamin D are optimal for preventing the disease—and whether the statistical associations reflect different gene pools rather than different levels of 25(OH)D. (Interestingly, Holick reported in the August 1988 issue of *The Journal of Clinical Endocrinology & Metabolism* that no previtamin D_3_ formed when human skin was exposed to sunlight on cloudless days in Boston, at 42.2°N, from November through February or in Edmonton, at 52°N, from October through March.)

“Scientific evidence on specific effects of vitamin D in preventing MS or slowing its progression is not sufficient,” says Alberto Ascherio, a nutritional epidemiologist at the Harvard School of Public Health. “Nevertheless, considering the safety of vitamin D even in high doses, there is no clear contraindication, and because vitamin D deficiency is very prevalent, especially among MS patients, taking vitamin D supplements and getting moderate sun exposure is more likely to be beneficial than not.”

As with MS, there appears to be a latitudinal gradient for type 1 diabetes, with a higher incidence at higher latitudes. A Swedish epidemiologic study published in the December 2006 issue of *Diabetologia* found that sufficient vitamin D status in early life was associated with a lower risk of developing type 1 diabetes. Nonobese mice of a strain predisposed to develop type 1 diabetes showed an 80% reduced risk of developing the disease when they received a daily dietary dose of 1,25(OH)D, according to research published in the June 1994 issue of the same journal. And a Finnish study published 3 November 2001 in *The Lancet* showed that children who received 2,000 IU vitamin D per day from 1 year of age on had an 80% decreased risk of developing type 1 diabetes later in life, whereas children who were vitamin D deficient had a fourfold increased risk. Researchers are now seeking to understand how much UVR/vitamin D is needed to lower the risk of diabetes and whether this is a factor only in high-risk groups.

There is also a connection with metabolic syndrome, a cluster of conditions that increases one’s risk for type 2 diabetes and cardiovascular disease. A study in the September 2006 issue of *Progress in Biophysics and Molecular Biology* demonstrated that in young and elderly adults, serum 25(OH)D was inversely correlated with blood glucose concentrations and insulin resistance. Some studies have demonstrated high prevalence of low vitamin D levels in people with type 2 diabetes, although it is not clear whether this is a cause of the disease or an effect of another causative factor—for example, lower levels of physical activity (in this case, outdoor activity in particular).

People living at higher latitudes throughout the world are at higher risk of hypertension, and patients with cardiovascular disease are often found to be deficient in vitamin D, according to research by Harvard Medical School professor Thomas J. Wang and colleagues in the 29 January 2008 issue of *Circulation*. “Although the exact mechanisms are poorly understood, it is known that 1,25(OH)D is among the most potent hormones for down-regulating the blood pressure hormone renin in the kidneys,” says Holick. “Moreover, there is an inflammatory component to atherosclerosis, and vascular smooth muscle cells have a vitamin D receptor and relax in the presence of 1,25(OH)D, suggesting a multitude of mechanisms by which vitamin D may be cardioprotective.”

To determine the potential link betwen sun exposure and the protective effect in preventing hypertension, Rolfdieter Krause of the Free University of Berlin Department of Natural Medicine and colleagues exposed a group of hypertensive adults to a tanning bed that emitted full-spectrum UVR similar to summer sunlight. Another group of hypertensive adults was exposed to a tanning bed that emitted UVA-only radiation similar to winter sunlight. After three months, those who used the full-spectrum tanning bed had an average 180% increase in their 25(OH)D levels and an average 6 mm Hg decrease in their systolic and diastolic blood pressures, bringing them into the normal range. In constrast, the group that used the UVA-only tanning bed showed no change in either 25(OH)D or blood pressure. These results were published in the 29 August 1998 issue of *The Lancet*. According to Krause, who currently heads the Heliotherapy Research Group at the Medical University of Berlin, a serum 25(OH)D level of at least 40 ng/mL should be adequate to protect against hypertension and other forms of cardiovascular disease (as well as cancers of the prostate and colon).

William Grant, who directs the Sunlight, Nutrition, and Health Research Center, a research and education organization based in San Francisco, suspects that sun exposure and higher 25(OH)D levels may confer protection against other illnesses such as rheumatoid arthritis (RA), asthma, and infectious diseases. “Vitamin D induces cathelicidin, a polypeptide that effectively combats both bacterial and viral infections,” Grant says. “This mechanism explains much of the seasonality of such viral infections as influenza, bronchitis, and gastroenteritis, and bacterial infections such as tuberculosis and septicemia.” For example, RA is more severe in winter, when 25(OH)D levels tend to be lower, and is also more prevalent in the higher latitudes. In addition, 25(OH)D levels are inversely associated with the clinical status of RA patients, and greater intake of vitamin D has been linked with lower RA risk, as reported in January 2004 in *Arthritis & Rheumatism*.

Some reports, including an article in the October–December 2007 issue of *Acta Medica Indonesiana*, indicate that sufficient 1,25(OH)D inhibits induction of disease in RA, collagen-induced arthritis, Lyme arthritis, autoimmune encephalomyelitis, thyroiditis, inflammatory bowel disease, and systemic lupus erythematosus. Nonetheless, interventional data are lacking for most autoimmune disorders and infectious diseases, with the exception of TB.

## How Much Is Enough?

Gilchrest points out a problem with the literature: “Everyone recommends something different, depending on the studies with which they are most aligned. One study reports an increased risk of prostate cancer for men with 25(OH)D levels above 90 ng/mL, for example.” In the June 2007 Lappe article, she notes, subjects in the control “high-risk” unsupplemented group had 25(OH)D levels of 71 nmol/L and the supplemented group had levels of 96 nmol/L.

Nevertheless, given the epidemiologic backdrop described above, there are now calls to rethink sun exposure policy or to promote vitamin D supplementation in higher-risk populations. Such groups include pregnant or breastfeeding women (these states draw upon a mother’s own reserves of vitamin D), the elderly, and those who must avoid the sun. Additionally, solely breastfed infants whose mothers were vitamin D deficient during pregnancy have smaller reserves of the nutrient and are at greater risk of developing rickets. Even in the sun-rich environment of the Middle East, insufficient vitamin D is a severe problem among breast-fed infants of women who wear a *burqa* (a traditional garment that covers the body from head to foot), as reported in the February 2003 *Journal of Pediatrics*.

Several recent reports indicate an increase in rickets particularly among breastfed black infants, though white babies also are increasingly at risk. A study in the February 2007 *Journal of Nutrition* concluded that black and white pregnant women and neonates in the northern United States are at high risk of vitamin D insufficiency, even when mothers take prenatal vitamins (which typically provide 100–400 IU vitamin D_3_). Studies by Bruce Hollis, director of pediatric nutritional sciences at the Medical University of South Carolina, and colleagues suggest that a maternal vitamin D_3_ intake of 4,000 IU per day is safe and sufficient to ensure adequate vitamin D status for both mother and nursing infant.

These days, most experts define vitamin D deficiency as a serum 25(OH)D level of less than 20 ng/mL. Holick and others assert that levels of 29 ng/mL or lower can be considered to indicate a relative insufficiency of vitamin D. Using this scale and considering various epidemiologic studies, an estimated 1 billion people worldwide have vitamin D deficiency or insufficiency, says Holick, who adds, “According to several studies, some forty to one hundred percent of the U.S. and European elderly men and women still living in the community [that is, not in nursing homes] are vitamin D deficient.” Holick asserts that a large number of infants, children, adolescents, and postmenopausal women also are vitamin D insufficient. “These individuals have no apparent skeletal or calcium metabolism abnormalities but may be at much higher risk of developing various diseases,” Holick says.

In the context of inadequate sunlight or vitamin D insufficiency, some scientists worry that the emphasis on preventing skin cancers tends to obscure the much larger mortality burden posed by more life-threatening cancers such as lung, colon, and breast cancers. Many studies have shown that cancer-related death rates decline as one moves toward the lower latitudes (between 37°N and 37°S), and that the levels of ambient UVR in different municipalities correlate inversely with cancer death rates there. “As you head from north to south, you may find perhaps two or three extra deaths [per hundred thousand people] from skin cancer,” says Vieth. “At the same time, though, you’ll find thirty or forty fewer deaths for the other major cancers. So when you estimate the number of deaths likely to be attributable to UV light or vitamin D, it does is not appear to be the best policy to advise people to simply keep out of the sun just to prevent skin cancer.”

To maximize protection against cancer, Grant recommends raising 25(OH)D levels to between 40 and 60 ng/mL. Research such as that described in Holick’s August 2006 *Journal of Clinical Investigation* article indicates that simply keeping the serum level above 20 ng/mL could reduce the risk of cancer by as much as 30–50%.

Cedric F. Garland, a medical professor at the University of California, San Diego, says that maintaining a serum level of 55–60 ng/mL may reduce the breast cancer rate in temperate regions by half, and that incidence of many other cancers would be similarly reduced as well. He calls this “the single most important action that could be taken by society to reduce the incidence of cancer in North America and Europe, beyond not smoking.” Moreover, these levels could be readily achieved by consuming no more than 2,000 IU/day of vitamin D_3_ at a cost of less than $20 per year and, unless there are contraindications to sunlight exposure, spending a few minutes outdoors (3–15 minutes for whites and 15–30 minutes for blacks) when the sun is highest in the sky, with 40% of the skin area exposed.

Holick, Vieth, and many other experts now make a similar daily recommendation: 4,000 IU vitamin D_3_ without sun exposure or 2,000 IU plus 12–15 minutes of midday sun. They say this level is quite safe except for sun-sensitive individuals or those taking medications that increase photosensitivity.

Gilchrest says some sunlight enters the skin even through a high-SPF sunscreen, so people can maximize their dermal vitamin D production by spending additional time outdoors while wearing protection. “Without the sunscreen, this same individual would be incurring substantially more damage to her skin but not further increasing her vitamin D level,” she says.

## Creating a Balanced Message

A growing number of scientists are concerned that efforts to protect the public from excessive UVR exposure may be eclipsing recent research demonstrating the diverse health-promoting benefits of UVR exposure. Some argue that the health benefits of UVB radiation seem to outweigh the adverse effects, and that the risks can be minimized by carefully managing UVR exposure (e.g., by avoiding sunburn), as well as by increasing one’s intake of dietary antioxidants and limiting dietary fat and caloric intake. Antioxidants including polyphenols, apigenin, curcumin, proanthocyanidins, resveratrol, and silymarin have shown promise in laboratory studies in protecting against UVR-induced skin cancer, perhaps through antimutagenic or immune-modulating mechanisms.

Central to the emerging debate is the issue of how to best construct public health messages that highlight the pros and cons of sun exposure in a balanced way. Such messages must necessarily take into account variations in skin pigmentation between groups and these groups’ differing susceptibilities to the dangers and benefits of sun exposure. Moreover, says Patricia Alpert, a nursing professor at the University of Las Vegas, age matters. “The elderly [have a] declining capacity to make vitamin D,” she says. “Many elderly, especially those living in nursing homes, are vitamin D deficient, [even] those living in areas considered to have adequate sunshine.”

Many experts are now recommending a middle-ground approach that focuses on modest sun exposures. Gilchrest says the American Academy of Dermatology and most dermatologists currently suggest sun protection in combination with vitamin D supplementation as a means of minimizing the risk of both skin cancer and internal cancers. Furthermore, brief, repeated exposures are more efficient at producing vitamin D. “Longer sun exposures cause further sun damage to skin and increase the risk of photo-aging and skin cancer, but do not increase vitamin D production,” she explains.

Lucas adds that people should use sun protection when the UV Index is more than 3. As part of Australia’s SunSmart program, “UV Alerts” are announced in newspapers throughout the country whenever the index is forecast to be 3 or higher. “Perhaps,” she says, “this practice should be extended to other nations as well.” U.S. residents can obtain UV Index forecasts through the EPA’s SunWise website (http://epa.gov/sunwise/uvindex.html).

In the near future, vitamin D and health guidelines regarding sun exposure may need to be revised. But many factors not directly linked to sun protection will also need to be taken into account. “Current observations of widespread vitamin D insufficiency should not be attributed only to sun protection strategies,” says Lucas. “Over the same period there is a trend to an increasingly indoor lifestyle, associated with technological advances such as television, computers, and video games.” She says sun-safe messages remain important—possibly more so than ever before—to protect against the potentially risky high-dose intermittent sun exposure that people who stay indoors may be most likely to incur.

## Serotonin, Melatonin, and Daylight

As diurnal creatures, we humans are programmed to be outdoors while the sun is shining and home in bed at night. This is why melatonin is produced during the dark hours and stops upon optic exposure to daylight. This pineal hormone is a key pacesetter for many of the body’s circadian rhythms. It also plays an important role in countering infection, inflammation, cancer, and auto-immunity, according to a review in the May 2006 issue of *Current Opinion in Investigational Drugs*. Finally, melatonin suppresses UVR-induced skin damage, according to research in the July 2005 issue of *Endocrine*.

When people are exposed to sunlight or very bright artificial light in the morning, their nocturnal melatonin production occurs sooner, and they enter into sleep more easily at night. Melatonin production also shows a seasonal variation relative to the availability of light, with the hormone produced for a longer period in the winter than in the summer. The melatonin rhythm phase advancement caused by exposure to bright morning light has been effective against insomnia, premenstrual syndrome, and seasonal affective disorder (SAD).

The melatonin precursor, serotonin, is also affected by exposure to daylight. Normally produced during the day, serotonin is only converted to melatonin in darkness. Whereas high melatonin levels correspond to long nights and short days, high serotonin levels in the presence of melatonin reflect short nights and long days (i.e., longer UVR exposure). Moderately high serotonin levels result in more positive moods and a calm yet focused mental outlook. Indeed, SAD has been linked with low serotonin levels during the day as well as with a phase delay in nighttime melatonin production. It was recently found that mammalian skin can produce serotonin and transform it into melatonin, and that many types of skin cells express receptors for both serotonin and melatonin.

With our modern-day penchant for indoor activity and staying up well past dusk, nocturnal melatonin production is typically far from robust. “The light we get from being outside on a summer day can be a thousand times brighter than we’re ever likely to experience indoors,” says melatonin researcher Russel J. Reiter of the University of Texas Health Science Center. “For this reason, it’s important that people who work indoors get outside periodically, and moreover that we all try to sleep in total darkness. This can have a major impact on melatonin rhythms and can result in improvements in mood, energy, and sleep quality.”

For people in jobs in which sunlight exposure is limited, full-spectrum lighting may be helpful. Sunglasses may further limit the eyes’ access to full sunlight, thereby altering melatonin rhythms. Going shades-free in the daylight, even for just 10–15 minutes, could confer significant health benefits.

## Other Sun-Dependent Pathways

The sun may be best known for boosting production of vitamin D, but there are many other UVR-mediated effects independent of this pathway.

**Direct immune suppression.** Exposure to both UVA and UVB radiation can have direct immunosuppressive effects through upregulation of cytokines (TNF-α and IL-10) and increased activity of T regulatory cells that remove self-reactive T cells. These mechanisms may help prevent autoimmune diseases.

**Alpha melanocyte-stimulating hormone (**α**-MSH).** Upon exposure to sunshine, melanocytes and keratinocytes in the skin release α-MSH, which has been implicated in immunologic tolerance and suppression of contact hypersensitivity. α-MSH also helps limit oxidative DNA damage resulting from UVR and increases gene repair, thus reducing melanoma risk, as reported 15 May 2005 in *Cancer Research*.

**Calcitonin gene-related peptide (CGRP).** Released in response to both UVA and UVB exposure, this potent neuropeptide modulates a number of cytokines and is linked with impaired induction of immunity and the development of immunologic tolerance. According to a report in the September 2007 issue of *Photochemistry and Photobiology*, mast cells (which mediate hypersensitivity reactions) play a critical role in CGRP-mediated immune suppression. This could help explain sunlight’s efficacy in treating skin disorders such as psoriasis.

**Neuropeptide substance P.** Along with CGRP, this neuropeptide is released from sensory nerve fibers in the skin following UVR exposure. This results in increased lymphocyte proliferation and chemotaxis (chemically mediated movement) but may also produce local immune suppression.

**Endorphins.** UVR increases blood levels of natural opiates called endorphins. Melanocytes in human skin express a fully functioning endorphin receptor system, according to the June 2003 *Journal of Investigative Dermatology*, and a study published 24 November 2005 in *Molecular and Cellular Endocrinology* suggests that the cutaneous pigmentary system is an important stress-response element of the skin.

## Research Challenges

Growing evidence of the beneficial effects of UVR exposure has challenged the sun-protection paradigm that has prevailed for decades. Before a sun-exposure policy change occurs, however, we need to know if there is enough evidence to infer a protective effect of sun exposure against various diseases.

Only through well-designed randomized clinical trials can cause-and-effect relationships be established. However, most sunlight-related epidemiologic research to date has relied on observational data that are subject to considerable bias and confounding. Findings from observational studies are far less rigorous and reliable than those of interventional studies. But interventional studies would need to be very large and carried out over several decades (since most UVR-mediated diseases occur later in life). Moreover, it is not at all clear when, over a lifetime, sun exposure/vitamin D is most important. So for now scientists must rely on the results of well-conducted observational analytic studies.

In sunlight-related research, there are two main exposures of interest: vitamin D status, which is measured by the serum 25(OH)D level; and personal UVR dose, which involves three fundamental factors: ambient UVR (a function of latitude, altitude, atmospheric ozone levels, pollution, and time of year), amount of skin exposed (a function of behavioral, cultural, and clothing practices), and skin pigmentation (with dark skin receiving a smaller effective dose to underlying structures than light skin).

When measuring sun exposure at the individual level, many scientists have relied on latitude or ambient UVR of residence. But these measures are fraught with uncertainties. “While ambient UVR varies, . . . so too do a variety of other possible etiological factors, including diet, exposure to infectious agents, temperature, and possibly even physical activity levels,” says Robyn Lucas, an epidemiologist at Australia’s National Centre for Epidemiology and Population Health. “Additionally, under any level of ambient UVR, the personal UV dose may vary greatly. In short, there is no real specificity for ambient UVR.”

Researchers also assess history of time in the sun at various ages, history of sunburns, dietary and supplemental vitamin D intake, and other proxy measures. Nonetheless, says Lucas, “there are drawbacks to inferring that a relationship with any proxy for the exposure of interest is a relationship with personal UV dose or vitamin D status.” On the bright side, she adds, our ability to accurately gauge an individual’s UV dose history has been enhanced with the use of silicone rubber casts of the back of subjects’ hands. The fine lines recorded by the cast provide an objective measure of cumulative sun damage.

## Figures and Tables

**Figure:**
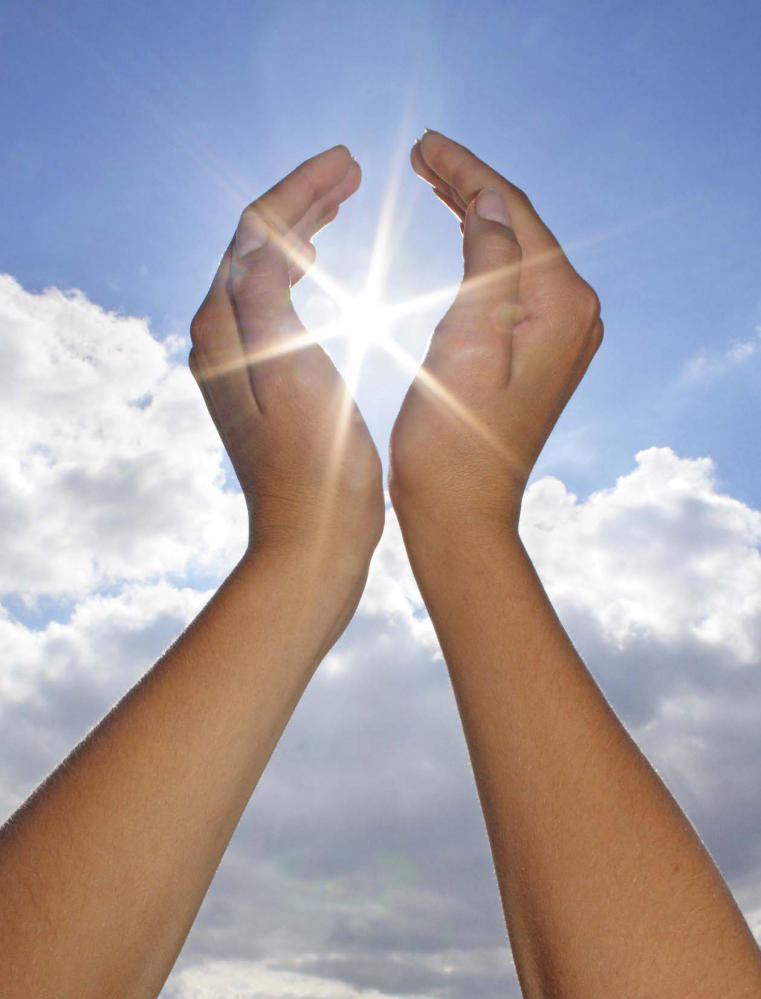


**Figure:**
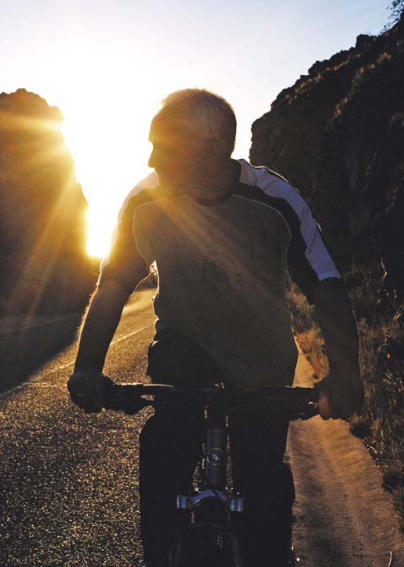
Personal UVB dose and risk of several types of cancer both depend in part on latitude of residence. These maps show a striking concordance between differential UVB dose across the United States and mortality rates of breast cancer among white women.

**Figure:**
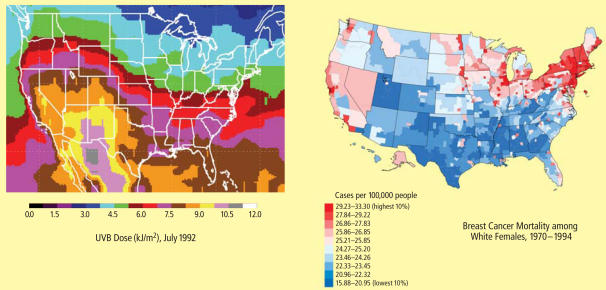


**Figure:**
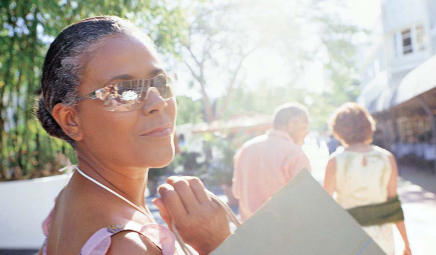


**Figure:**