# ERRATUM

**Published:** 2008-05

The April 2008 Focus article [ Environ Health Perspect 116:A160–A167 (2008)] includes a misprint. The first paragraph under the subhead “How Much Is Enough?” should read: “Gilchrest points out a problem with the literature: ‘Everyone recommends something different, depending on the studies with which they are most aligned. One study reports an increased risk of prostate cancer for men with 25(OH)D levels above 90 ng/mL, for example [in contrast with the idea that more vitamin D is more protective against cancer].’” *EHP* regrets the error.

